# Coexistence and different determinants of posttraumatic stress disorder and posttraumatic growth among Chinese survivors after earthquake: role of resilience and rumination

**DOI:** 10.3389/fpsyg.2015.01043

**Published:** 2015-08-04

**Authors:** Kaijun Wu, Yuqing Zhang, Zhengkui Liu, Peiling Zhou, Chuguang Wei

**Affiliations:** Key Laboratory of Mental Health, Institute of Psychology, Chinese Academy of Sciences, University of Chinese Academy of SciencesBeijing, China

**Keywords:** PTSD, PTG, resilience, rumination, SEM

## Abstract

Posttraumatic stress disorder (PTSD) and posttraumatic growth (PTG) are two different outcomes that may occur after experiencing traumatic events. Resilience and rumination are two important factors that determine the development of these outcomes after trauma. We investigated the association between these two factors, PTSD and PTG, among Chinese survivors in an earthquake. With a convenience sample of 318 survivors from earthquake, we measured trauma exposure, PTSD, PTG, resilience, and rumination (Impact of Event Scale-Revised, Posttraumatic Growth Inventory, 10 item Connor–Davidson Resilience Scale, Ruminative Response Scale). Then we used bivariate correlation and structural equation modeling to examine the structure of relations among these factors. Results showed that resilience and reflective rumination have a positive effect on PTG (β = 0.32, *p* < 0.001; β = 0.17, *p* = 0.049). Earthquake exposure, brooding rumination and depressed-related rumination are related with higher level of PTSD (β = 0.10, *p* = 0.021; β = 0.33, *p* < 0.001; β = 0.36, *p* < 0.001). The findings suggest distinct determinants of the negative and positive outcomes, and this may provide better understanding about the risk and protective factors for traumatic reactions.

## Introduction

Two common but different outcomes may occur among survivors of traumatic events, such as natural disasters (Dekel et al., 2012). One is a pathogenic outcome, which includes anxiety, depression, acute stress disorder (ASD), and posttraumatic stress disorder (PTSD). People who suffered from traumatic events may display several negative symptoms, such as intrusive thoughts, avoidance of trauma-related events, hyper-arousal, and sleep disorder. Owing to the developments in positive psychology, researchers are often focusing more on the salutogenic outcomes. Salutogenic means being good for health, and this word originates from salutogenesis—the origins of health (Antonovsky, 1979). The most common salutogenic outcome is posttraumatic growth (PTG). PTG refers to the positive psychological changes and developments through the struggle with the challenges of life after traumatic events (Tedeschi and Calhoun, 2004). PTG comprises five components, including relating to others, new possibilities, personal strength, spiritual change, and appreciation of life (Tedeschi and Calhoun, 1996).

When considering about the relationship between PTSD and PTG, some research shows that they are positively related (Morris et al., 2005; Taku et al., 2008; Bensimon, 2012). This finding indicates that these two trauma manifestations could coexist and are not mutually exclusive. According to the multifactorial psychosocial framework of posttraumatic adjustment (Joseph and Williams, 2005), personality influences coping, emotional state and appraisal which constitute PTSD reactions, and these factors in turn influence personality which constitutes PTG (Joseph and Linley, 2008). In the PTG development model (Tedeschi and Calhoun, 2004), PTG occurs only when the trauma is strong enough such that survivors start to engage in meaning-making of the event. As measured by the Impact of Event Scale-Revised (IES-R), intrusive thoughts are considered as a kind of cognitive processing, which can help survivors understand and work through the consequences of disasters (Helgeson et al., 2006). Therefore, experiencing some PTSD symptoms, such as intrusive thoughts, and a period of consideration of traumatic events are necessary for the development of PTG. These two models help to explain the relative independence between PTSD and PTG. However, the positive relationship between PTSD and PTG is not replicated in some other studies. A longitudinal study carried out with sexual assault survivors indicated that PTSD and PTG are negatively associated. However, other studies showed that the relationship between PTG and PTSD is curvilinear (inverted-U; Frazier et al., 2001; Levine et al., 2008), which supposes that maybe a moderate level of PTSD promotes the development of PTG better. The non-conformity of the relationship between PTSD and PTG requires further research.

Another focus of this research is resilience, for which there are two different definitions in the literature. One is the ability to cope with and adapt to the changes after threatening or challenging situations (Agaibi and Wilson, 2005; Bonanno et al., 2006). The other is the phenomenon that people have suffered from traumatic events without developing any PTSD symptom (Lepore and Revenson, 2006; Levine et al., 2009). In this study, we define resilience as a collection of abilities and coping strategies instead of a phenomenon, because we intend to explore how these abilities can affect the traumatic outcomes. Resilience helps people to recover from traumatic events and extreme stress (Wilson and Drozdek, 2004). As a trait, resilience is negatively related to posttraumatic distress (Connor and Davidson, 2003; Pietrzak et al., 2010). This relationship indicates that resilience can reduce pathogenic reactions after suffering from traumatic events, which coincides with the model built by Agaibi and Wilson (2005). This model indicates that resilience related to personalities and coping styles affects thinking and perception, which modulates PTSD symptoms. Studies exploring the relationship between resilience and PTG are inconsistent. A study in Israel (Bensimon, 2012) indicates that resilience and PTG were positively related because these two could coexist as salutogenic factors when facing traumatic events (Lepore and Revenson, 2006). Resilient-coping strategies and cognitive processes could promote growth after traumatic events, as supported by the model developed by Calhoun and Tedeschi (2004). In contrast, another study (Levine et al., 2009) showed that PTG and resilience were negatively related, i.e., people with high levels of resilience might have the ability to protect themselves from threatening or negative changes when facing traumatic events, so resilience outcomes may provide little opportunity for growth (Westphal and Bonanno, 2007). However, PTG has a positive correlation with both PTSD and resilience. Considering resilience is negatively related to PTSD, the negative correlation between PTG and resilience seems incompatible.

In several models describing the development of PTG, rumination is identified as another important factor that is part of the cognitive process of psychological changes after traumatic events. According to the response styles theory (Nolen-Hoeksema, 1991), rumination refers to the repetitive passive and self-focused responses to the harm resulting from traumatic events, which focuses on traumatic symptoms as well as the possible causes and consequences of these symptoms. Rumination was previously believed to be connected with negative traumatic outcomes because ruminative people remain focusing on the traumatic events and the feelings instead of taking action. The notion of rumination being positively related to distress or PTSD symptoms and being considered as one of the most important predictors of PTSD was supported by several studies (Nolen-Hoeksema, 2000; Murray et al., 2002; Sarin et al., 2005) that consider rumination as a whole concept.

In recent years, researchers have realized that the construct of rumination is multidimensional and needs a broader view. According to Treynor et al. (2003), rumination includes three different factors, namely, brooding, reflection, and depression-related rumination. Brooding rumination is characterized by a repetitive, intrusive, and passive consideration of traumatic experience or negative emotions. Brooding people always compare the current situation with an unachieved standard, and this comparison may prevent them from exerting efforts to overcome difficulties. On the contrary, reflective rumination, as a relatively good form of rumination, represents a more deliberate and positive consideration that focuses on the struggle with traumatic events. Recent studies (Taku et al., 2008; Taku et al., 2009; Stockton et al., 2011) that focus on brooding and reflective rumination showed the differences between the relationship of these two rumination factors with PTSD and PTG. For instance, brooding rumination was positively related to PTSD symptoms and distress, whereas reflective rumination was associated with a higher level of salutogenic outcomes and lower level of distress. Depression-related rumination is the third dimension, which contains items about depressive symptoms. These items are similar to Beck Depression Inventory items (e.g., think about how passive and unmotivated you feel, think about how alone you feel). The comorbidity between PTSD and depression is supported by many studies (Bleich et al., 1997; Campbell et al., 2007; O’Donnell et al., 2014), so we assume that depression-related rumination is positively associated with PTSD.

Resilience and rumination are two important factors in the development of psychological changes after traumatic events, according to the PTG model and previous research. However, only a few studies have examined the role of resilience and different dimensions of rumination as determinants of PTSD and PTG in one model. Based on the data obtained from the survivors of an earthquake in China, the purpose of this study is to examine the association between PTSD and PTG, and the effect of resilience, the three factors of rumination (brooding, reflection and depressed-related) on PTG and PTSD. With this research, we hope to explore the different effects of resilience and rumination on PTSD and PTG, which may help to provide empirical support for psychological intervention after traumatic events like natural disasters.

**FIGURE 1:**
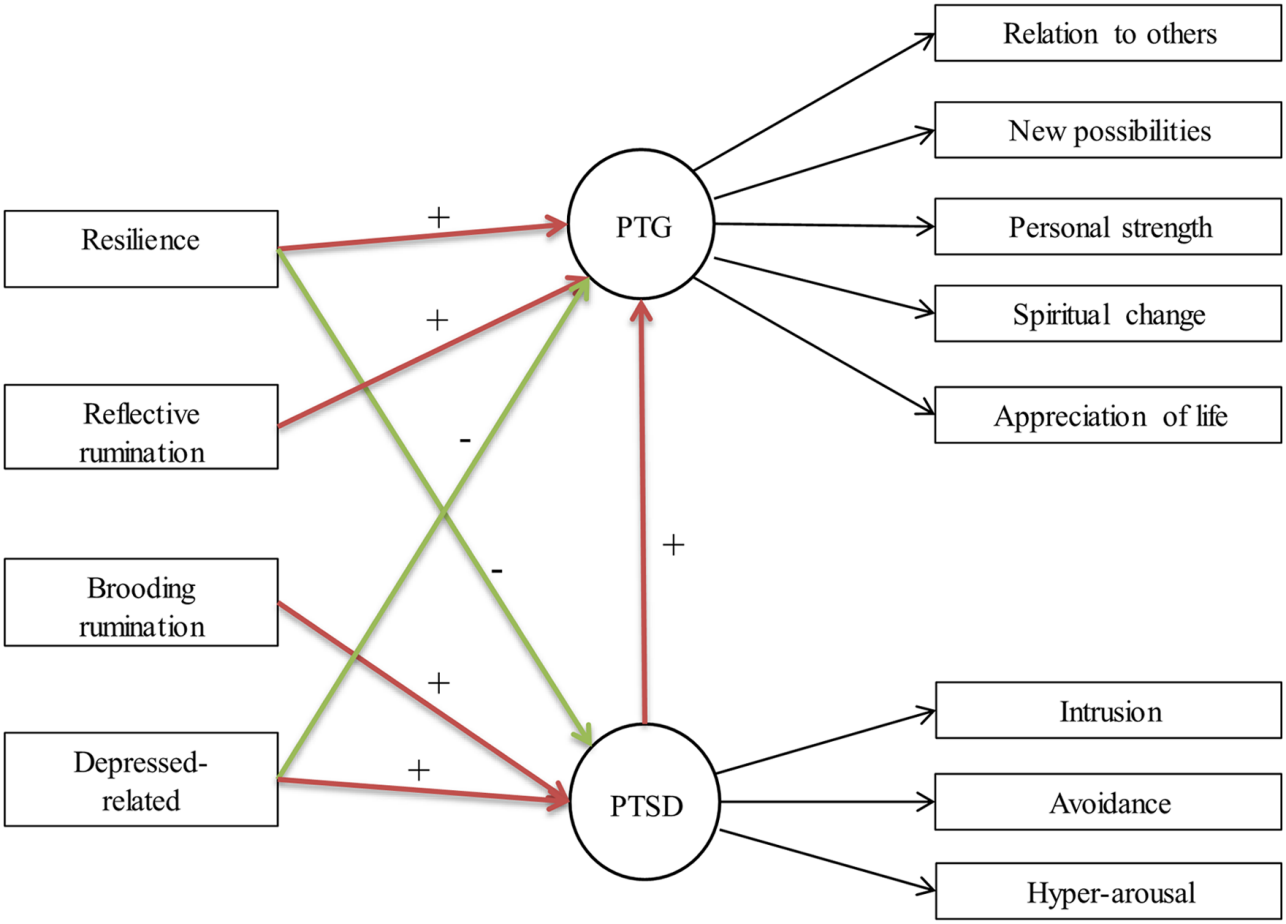
**Hypothetic model of the relationships among posttraumatic stress disorder (PTSD), posttraumatic growth (PTG), resilience, and rumination**.

Based on the literature above, the hypotheses are as follows (**Figure 1**):

(1) PTSD will be positively related to PTG.
(2) PTSD will be negatively associated with resilience, and positively associated depression-related rumination and brooding rumination;
(3) PTG will be positively associated with resilience and reflective rumination, and negatively associated with depress-related rumination.

## Materials and Methods

### Participants

Three hundred and fifty survivors in Yiliang county, China were recruited about 3 months after the 2012 earthquake. Every participant was in the earthquake region and experienced the earthquake when it happened. Thirty-two were excluded because of uncompleted questionnaire items or because of being younger than 16 years-old. Ten of the 318 survivors didn’t report their demographic information. Considering this part wouldn’t affect the result of this study, we did not exclude the 10 questionnaires. So 318 participants were included in the final analysis.

### Procedure

In the study, the participants were living either in their own homes or in relief tents put up by the government. The questionnaires were distributed by a master student and a local volunteer. Both of them were trained by experienced psychologyl professors about how to explain the informed consent procedure and instructions of the questionnaire. They visited the survivors from door to door to recruit participants by asking whether they were willing to answer a set of questionnaires on their mental health after experiencing the earthquake. The questionnaire was completed in the participants’ own houses or relief tents with the company of researchers, so that when they had any question, they could get help. Data collection was conducted approximately 3 months after the earthquake, and lasted for 3 weeks (from December 19, 2012 to January 6, 2013). Written informed consent from the participants was obtained prior to their answering of the questionnaire. Eleven percent of the participants were high school students who were from 16 to 17. Thus, before the survey, we also obtained verbal consent from their parents. We explained the purpose and procedures of our investigation and presented the printed informed consent to the parents. With their approval for the investigation, we explained the consent to their children and let the children sign the written informed consent. We did not get written consent from parents since they were not considered participants. It would be difficult to make them understand that signing the forms lead in the survey to no other commitments and consequences apart from contributing to the current research. We aimed not to prompt potential worries among these parents. Ethical review was approved by the ethics committee of Institute of Psychology, Chinese Academy of Sciences.

### Measures

A demographic questionnaire was included to obtain participants’ personal information including age, marital status, education level, and monthly family income.

A specially developed questionnaire was adopted to assess the trauma exposure of the survivors during the earthquake. This questionnaire contained six items. Participants were asked whether they had (a) been trapped in the ruins of collapsed buildings; (b) lost their home as it collapsed or being severely damaged; (c) witnessed the death of someone; (d) suffered from physical injures; and (e) experienced bereavement. The participants answered ‘yes’ or ‘no’ to each item (yes = 1 and no = 0). The sum of the responses of these six items (range from 0 to 5) would be the total trauma exposure score. A higher score implied greater exposure to traumatic incidents during the earthquake.

Posttraumatic stress disorder was assessed by the IES-R (Weiss and Marmar, 1997). The tool is a 22-item inventory that contains three clusters, namely, intrusion, avoidance, and hyper-arousal symptoms. Participants were asked to indicate whether they had symptoms in the previous week on a five-point scale ranging from 0 (not at all) to 4 (nearly all the time). The Chinese version of IES-R was verified by Huang et al. (2006). The Cronbach’s alpha of IES-R in this study was 0.939 for the overall scale, and 0.900, 0.821, and 0.802 for the intrusion, avoidance, and hyper-arousal subscales, respectively.

Posttraumatic growth was measured by the Posttraumatic Growth Inventory (PTGI) developed by Tedeschi and Calhoun (1996), which measures perceived positive changes after suffering from traumatic events. PTGI is a 21-item self-reported inventory with five subscales, which are relation to others, new possibilities, personal strengths, spiritual change, and appreciation of life. The Chinese version of PTGI was developed through translation and back-translation. This version is a six-point scale ranging from 0 (I did not experience this change after the traumatic event) to 5 (I experienced this change to a very great degree after the traumatic event). In this study, the participants were asked to estimate the positive changes they experienced after the Yiliang earthquake. The Cronbach’s alpha of the Chinese version of PTGI in the current study was 0.86.

Resilience was measured by the 10-item Connor–Davidson Resilience Scale (CD-RISC), which was developed by Campbell-Sills and Stein (2007) according to the original 25-item and five-dimension CD-RISC (Connor and Davidson, 2003). This 10-item scale measures personal qualities that enable people to thrive after exposure to trauma (e.g., able to adapt to change, can handle unpleasant feelings). Each item is rated on a five-point scale from 0 (not true at all) to 4 (almost always true). The English version of 10-item CD-RISC has a good internal consistency (Cronbach’s α = 0.85), and a great structure validity in terms of goodness-of-fit scores. The Chinese version of the 10-item CD-RISC was validated in another Chinese survivor sample affected by earthquake (Wang et al., 2010). The Cronbach’s alpha in this study was 0.87.

Rumination was measured by the Ruminative Response Scale (RRS), which is a subscale of the Response Styles Questionnaire (RSQ; Nolen-Hoeksema and Morrow, 1991). The RRS includes 22 items that describe the response to depression or other symptoms arouse from traumatic events. Each item is rated on a four-point scale from 1 (almost never) to 4 (almost always). RRS is composed of three dimensions, which are brooding, reflection, and depression-related. Brooding is interpreted as a passive comparison of current situation with an unachieved standard (e.g., think “What am I doing to deserve this?” think “Why do I have problems other people don’t have?”). Reflection means a positive consideration of coping with traumatic events(e.g., analyze recent events to try to understand why you are depressed, analyze your personality to try to understand why you are depressed). Depression-related contained some symptoms of depression (e.g., think about how passive and unmotivated you feel, think about how alone you feel). This scale has a good validity and a stable structure to support the multidimensional nature of rumination (Treynor et al., 2003; Schoofs et al., 2010). The Chinese version of RRS has been widely used in adolescent samples (Lo et al., 2008; Ng and Bhugra, 2008). The Cronbach’s alpha of RRS in this study was 0.94 for the whole scale, and 0.78, 0.78, and 0.90 for the brooding, reflection, and depression-related subscales, respectively.

### Data Analysis

The analysis proceeded in two steps. In the first step, initial analysis, descriptive statistics for the scales, independent sample *t*-test, Pearson’s correlation analyses and analyses of variance (ANOVA) analyses were conducted using SPSS. In the second step, structural equation modeling was conducted by using AMOS 6 (version 1.0) to test the proposed model of PTSD, PTG, resilience and rumination. Resilience, reflective, brooding, and depression-related ruminations were entered as observed variables in the SEM. PTSD is a latent variable represented by intrusion, avoidance, and hyper-arousal symptoms, whereas PTG, and is represented by relation to others, new possibilities, personal strengths, spiritual change, and appreciation of life. NFI, CFI, GFI, RMSEA, and SRMR were used to assess the model fitness according to the guidelines for SEM (Bentler, 2007).

## Results

### Study Participants

**Table 1** shows the socio-demographic information and self-report measure scores (10 of the 318 subjects did not report their demographic information). Independent sample *t*-tests indicated that compared to female participants, male participants reported more PTG [*t*_(306)_ = 2.44, *p* = 0.015]. Pearson’s correlation analyses results showed that participants in older age tended to have higher level of PTSD symptoms [*r*_(306)_ = 0.24, *p* < 0.001]. ANOVAs indicated that married ones and those who didn’t finish high school education reported higher level of PTSD symptoms [*F*_(3,304)_ = 4.21, *p* = 0.006; *F*_(4,303)_ = 9.46, *p* < 0.001], and the uneducated reported less PTG [*F*_(4,303)_ = 4.22, *p* = 0.001].

**Table 1:** Descriptive statistics of the study measures.

Variable	
Age in years ± SD	28.60 ± 12.40
Gender, N (%)	
Male	142 (44.6)
Female	166 (52.2)
Marital status, N (%)	
Single	166 (52.2)
Married	136 (42.8)
Divorced or separated	3 (0.09)
Widowed	3 (0.09)
Ethnicity, N (%)	
Han national	247 (77.7)
Minor nationals	61 (19.2)
Education, N (%)	
Illiteracy	21 (6.6)
Primary level	39 (12.3)
Secondary school level	16 (5.0)
High school level	146 (45.9)
University degree or higher	85 (26.7)
Earthquake exposure, *M* ± SD	0.70 ± 0.80
PTSD, *M* ± SD	38.02 ± 19.18
Intrusion	15.24 ± 7.87
Avoidance	12.58 ± 6.63
Hyper-arousal symptoms	10.19 ± 6.45
PTG, *M* ± SD	64.59 ± 5.17
Relation to others	22.96 ± 5.69
New possibilities	13.09 ± 4.84
Personal strength	14.19 ± 4.15
Spiritual change	4.14 ± 2.76
Appreciation of life	10.22 ± 2.76
Relsilience, *M* ± SD	34.84 ± 7.49
Rumination, *M* ± SD	45.77 ± 13.44
Reflection	10.18 ± 3.25
Brooding	10.53 ± 3.47
Depressed-related	25.07 ± 7.90

*PTSD, total score of Impact of Event Scale-Revised; PTG, total score of posttraumatic growth inventory; Resilience, total score of 10-item Connor–Davidson Resilience Scale; Rumination, total score of Ruminative response scale (RRS).*

### Bivariate Correlations

**Table 2** presents the Pearson bivariate correlations between the scales and subscales. The examination of **Table 2** shows that PTSD is positively associated with PTG, rumination, and the three subscales of rumination. Earthquake exposure is positively associated with PTSD and PTG. Moreover, PTG is positively associated with total rumination, reflective, and brooding ruminations, and resilience. However, the correlations between PTSD and resilience, and between PTG and depression-related rumination, are not significant.

**Table 2:** Correlations of Impact of Event Scale-Revised (IES-R), Posttraumatic Growth Inventory (PTGI), 10-item Connor–Davidson Resilience Scale (CD-RISC), and RRS.

	1	2	3	4	5	6	7
(1) PTSD	1.00						
(2) PTG	0.15^∗∗^	1.00					
(3) Earthquake exposure	0.22^∗∗^	0.14^∗^	1.00				
(4) Resilience	-0.11	0.28^∗∗^	0.04	1.00			
(5) Rumination	0.64^∗∗^	0.13^∗^	0.19^∗∗^	-0.05	1.00		
(6) Reflection	0.49^∗∗^	0.17^∗∗^	0.15^∗∗^	0.07	0.84^∗∗^	1.00	
(7) Brooding	0.61^∗∗^	0.17^∗∗^	0.20^∗∗^	-0.05	0.89^∗∗^	0.70^∗∗^	1.00
(8) Depressed-related	0.62^∗∗^	0.08	0.17^∗∗^	-0.09	0.96^∗∗^	0.71^∗∗^	0.79^∗∗^

*df = 316, ^∗^*p* < 0.05, ^∗∗^*p* < 0.01.*

### Structural Equation Model

Structural equation modeling was conducted with AMOS and used maximum likelihood estimation with the covariance matrix. *A priori* theoretical model based on the hypotheses of this study was tested. Since trauma exposure maybe a confounding risk factor of PTSD and PTG (Linley and Joseph, 2004), which is also supported by the result of bivariate correlation in this study, we added earthquake exposure as an observed variable in our model. The result showed that the fit-to-data is acceptable: *X*^2^ = 127.26, df = 51, *X*^2^/df = 2.50, RMSEA = 0.069, RMSEA 90% fit indices 0.054–0.084, SRMR = 0.057, NFI = 0.94, CFI = 0.96, and GFI = 0.94.

**FIGURE 2:**
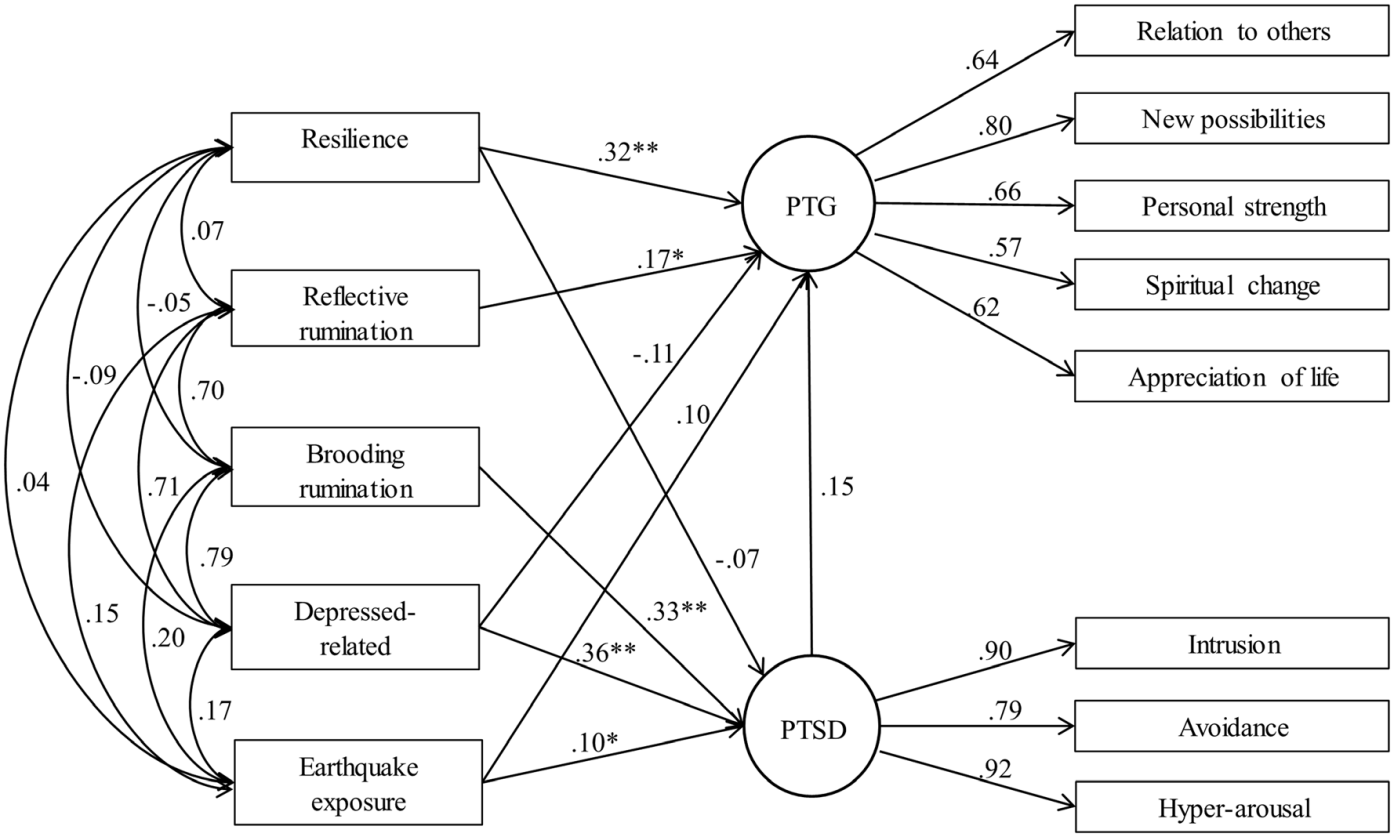
**Structural equation model of PTSD, PTG, resilience, and rumination.** ^∗^*p* < 0.05, ^∗∗^*p* < 0.01.

**Figure 2** shows the path estimates. Resilience and reflective rumination are related with higher level of PTG (β = 0.32, *p* < 0.001; β = 0.17, *p* = 0.049). Earthquake exposure, brooding rumination and depressed-related rumination are related with higher level of PTSD (β = 0.10, *p* = 0.021; β = 0.33, *p* < 0.001; β = 0.36, *p* < 0.001). However, neither the path from resilience to PTSD nor the path from depressed-related rumination to PTG is significant.

## Discussion

This study examined the role of resilience and the different factors of rumination in traumatic outcomes based on a sample of survivors of a natural disaster in China. According to the results, earthquake exposure was a negative predictor of PTSD symptoms, which means severe trauma exposure may lead to a higher level of PTSD symptoms. In addition, PTSD was shown to result from brooding rumination and depression-related rumination. PTG appeared to be associated with higher levels of resilience and reflective rumination, which indicates that positive and effective cognitive process could be helpful to the development of PTG. These results suggest that examining different types of rumination separately is necessary. However, the path from PTSD to PTG was not significant, neither was the path from resilience to PTSD.

Since the bivariate correlation showed that PTG and PTSD was positively correlated (*r* = 0.15, *p* < 0.01), the non-significant path in SEM maybe resulted from the low level of trauma exposure. Though every participant in this study experienced the disaster, only more than half of them suffered from severe impact, like severe physical injury, bereavement, or being trapped. The other participants were faced with property loss or house damage, and these loss were less destructive to survivors’ life after earthquake. Trauma exposure may facilitate the development of PTSD symptoms (Linley and Joseph, 2004), and the curvilinear relationship between PTSD and PTG indicates that moderate level of PTSD promotes the development of PTG better (Frazier et al., 2001; Levine et al., 2008). Thus, The low level of trauma exposure may weakened the relationship between PTSD and PTG.

The results showed that resilience was positively associated with PTG. This finding is consistent with the findings of previous research (Nishi et al., 2010; Bensimon, 2012). In this study, resilience refers to the cluster of positive traits, which includes character, personality and coping strategy according to the 10-item CDRS we used. Hence, this conclusion makes it easier to understand that resilience and PTG can coexist as two salutogenic factors (Lepore and Revenson, 2006). However, the relationship between PTSD and resilience was not significant in the present research, which is not consistent with previous studies. Many researches show that a higher level of resilience could help to reduce the likelihood of PTSD (Bensimon, 2012; Zerach et al., 2012; Ssenyonga et al., 2013; Streb et al., 2014; Besser et al., 2015). However, none of these used natural disaster survivors as subjects. Inaddition, some used different inventories to measure resilience, as Resilience Scale (RS-11) and The Life Orientation Test-Revised (LOT-R), and some of them defined resilience as the absence of posttraumatic symptoms instead of a collection of abilities. The differences of participants, definition, and measurements may affect the relationship between resilience and PTSD. So the effect of resilience on PTSD among natural disaster survivors may need further study.

Consistent with previous research (Morris et al., 2005; Taku et al., 2008, 2009), the results in the present study revealed that different kinds of rumination had varying contributions to the outcomes of traumatic events. As a deliberated and positive coping-related introspection, reflective rumination can help promoting the development of positive life changes after traumatic events, as supported by the model developed by Calhoun and Tedeschi (2004). This model showed that PTG was resulted from constructive cognitive processes. On the contrary, as a series of repetitive, intrusive, and negative cognitive process, brooding rumination might contribute to the experience of distress symptoms. These findings indicated that cognitive behavior interventions, which aimed at helping people to build reflective rumination instead of brooding rumination, are beneficial to survivors of traumatic events, and could promote positive life changes and reduce pathogenic symptoms (Chan et al., 2011; Rajandram et al., 2011). The results showed that depression-related rumination had a positive relationship with PTSD and a negative relationship with PTG. Although many studies have realized the necessity to separate different kinds of rumination, none of them had focused on the third part. Since depression-related rumination in RRS is measured by items related to depressive or anxious symptoms, its relationship with PTSD and PTG is easy to understand due to the overlapping between depression-related rumination and PTSD.

The present findings give rise to the suggestion that pathogenic and salutogenic traumatic outcomes have different relationships to cognitive factors as resilience and rumination. Even different dimensions of rumination may have separate effects on different traumatic outcomes. When natural disasters happen, psychological measurement of resilience and rumination for survivors may be necessary, because this is helpful for evaluating survivors’ anti-pressure ability. When screening high-risk group of PTSD participants, the score of resilience and rumination can also be considered. Therefore, practical psychological intervention about developing people’s ability to cope with and adapt to the changes after threatening or challenging situations may help to reduce PTSD symptoms and promote more PTG.

However, we acknowledge several limitations. First, convenience sampling of natural disaster survivors was employed. Thus, the sample did not represent the broader population with different traumatic events and cultural backgrounds. This limitation necessitates a replication of the study on larger groups of survivors from different kinds of traumatic events. Second, the present study was cross-sectional, and the measurements were all self-reported inventories, which might have weakened the potential strength of the conclusions. Therefore, this limitation indicates the need for a longitudinal study based on multiple-measures of PTSD, PTG, resilience, and rumination, which can affirm the causal relationship between these variables and help to understand the phenomena associated with PTG and PTSD. Third, the trauma exposure level in this study was relatively low, since the criteria we chose were quite severe. This would weaken the relationship between PTSD and PTG. The last, resilience and rumination were not the only two effect factors of traumatic outcomes, and other factors, such as social support may also affect the development of PTG and PTSD (Dirik and Karanci, 2008; Prati and Pietrantoni, 2009). Therefore, further research engaged with multi-aspect effect factors is required to explore the mechanism of the development of traumatic outcomes.

## Author Contributions

KW managed the data collection, undertook the statistical analysis, finished the first draft of the manuscript, and revised the draft for details. YZ designed the study and wrote the protocol, undertook the statistical analysis, and revised the draft for important intellectual content. ZL designed the study, wrote the protocol and revised the draft for important intellectual content. PZ helped to answer the comments and revised the draft for statistical issues. CW helped to answer the comments and revised the draft for grammar.

## Conflict of Interest Statement

The authors declare that the research was conducted in the absence of any commercial or financial relationships that could be construed as a potential conflict of interest.
